# Efficacy of an alcohol-focused intervention for improving adherence to antiretroviral therapy (ART) and HIV treatment outcomes – a randomised controlled trial protocol

**DOI:** 10.1186/1471-2334-14-500

**Published:** 2014-09-12

**Authors:** Charles DH Parry, Neo K Morojele, Bronwyn J Myers, Connie T Kekwaletswe, Samuel OM Manda, Katherine Sorsdahl, Gita Ramjee, Judith A Hahn, Jürgen Rehm, Paul A Shuper

**Affiliations:** Alcohol, Tobacco and Other Drug Research Unit, Medical Research Council, Cape Town, South Africa; Department of Psychiatry, University of Stellenbosch, Cape Town, South Africa; Alcohol, Tobacco and Other Drug Research Unit, Medical Research Council, Pretoria, South Africa; School of Public Health, University of the Witwatersrand, Johannesburg, South Africa; Department of Psychiatry and Mental Health, University of Cape Town, Cape Town, 7700 South Africa; Biostatistics Unit, Medical Research Council, Pretoria, South Africa; Alan J Flisher Centre for Public Mental Health, Department of Psychiatry, University of Cape Town Cape Town, South Africa; HIV Prevention Research Unit, Medical Research Council, Durban, South Africa; Department of Epidemiology and Population Health, London School of Hygiene & Tropical Medicine, London, United Kingdom; Department of Medicine, University of California San Francisco, San Francisco, CA USA; Social and Epidemiological Research Department, Centre for Addiction and Mental Health, Toronto, ON Canada; Dalla Lana School of Public Health, University of Toronto, Toronto, ON Canada; Technische Universität Dresden, Klinische Psychologie & Psychotherapie, Dresden, Germany; Center for Health, Intervention, and Prevention, University of Connecticut, Storrs, CT USA

**Keywords:** HIV/AIDS, Alcohol, South Africa, Randomised controlled trial, Brief intervention

## Abstract

**Background:**

Little research has examined whether alcohol reduction interventions improve antiretroviral therapy (ART) adherence and HIV treatment outcomes. This study assesses the efficacy of an intervention for reducing alcohol use among HIV patients on ART who are hazardous/harmful drinkers. Specific aims include adapting a blended Motivational Interviewing (MI) and Problem Solving Therapy (PST) intervention for use with HIV patients; evaluating the efficacy of the intervention for reducing alcohol consumption; and assessing counsellors’ and participants’ perceptions of the intervention.

**Methods/Design:**

A randomised controlled trial will evaluate the intervention among ART patients in public hospital-based HIV clinics in Tshwane, South Africa. We will recruit patients who are HIV-positive, on ART for at least 3 months, and classified as harmful/hazardous drinkers using the AUDIT-3. Eligible patients will be randomly assigned to one of three conditions. Patients in the experimental group will receive the MI-PST intervention to reduce harmful/hazardous alcohol use. Patients in the equal-attention wellness intervention group will receive an intervention focused on addressing health risk behaviours. Patients in the control condition will receive treatment as usual. Participants will complete an interviewer-administered questionnaire at baseline and 3, 6 and 12 months post-randomisation to assess alcohol consumption, ART adherence, physical and mental health. We will also collect biological specimens to test for recent alcohol consumption, CD4 counts and HIV RNA viral loads. The primary outcome will be reduction in the volume of alcohol consumed. Secondary outcomes include reduction in harmful/hazardous use of alcohol, reduction in biological markers of drinking, increase in adherence rates, reductions in viral loads, and increases in CD4 T-cell counts. A process evaluation will ascertain counsellors’ and participants’ perceptions of the acceptability and effectiveness of the interventions.

**Discussion:**

We have obtained ethical approval and approval from the study sites and regional and provincial health departments. The study has implications for clinicians, researchers and policy makers as it will provide efficacy data on how to reduce harmful/hazardous alcohol consumption among HIV patients and will shed light on whether reducing alcohol consumption impacts on HIV treatment adherence and other outcomes.

**Trial registration:**

Pan African Clinical Trials Register Number: PACTR201405000815100.

**Electronic supplementary material:**

The online version of this article (doi:10.1186/1471-2334-14-500) contains supplementary material, which is available to authorized users.

## Background

The consumption of alcohol has been closely investigated within the context of HIV [1]. Data suggests that hazardous alcohol use directly contributes to the acquisition and transmission of HIV as well as to antiretroviral therapy (ART) non-adherence, the worsening of HIV disease, progression to AIDS, and mortality [2–4]. Not only does South Africa have high numbers of persons who are HIV positive and on ART, but it also has one of the highest levels of per capita alcohol consumption *per drinker* globally and one of the highest levels of heavy episodic drinking globally among men and women [5].

ART is essential for improving and maintaining physical health, reducing HIV viral load, and reducing morbidity and mortality among people living with HIV and AIDS (PLWHA) [6]. Sub-optimal adherence to ART regimens can result in the development of resistance to ART, and ultimately poorer treatment outcomes, and mortality [7–10]. Alcohol consumption is a concern in relation to ART as alcohol may impact the cognitive processes necessary to maintain adequate adherence [11]. Furthermore, beliefs that ART medications should not be taken while consuming alcohol may cause drinkers to fail to adhere to treatment [12–15]. Hendershot et al.’s meta-analysis found that drinkers were 50%-60% as likely to be adherent as non-drinkers [16]. This effect was amplified for problem drinkers, who were only 47% as likely to be adherent as non-problem drinkers or abstainers. In addition, missed ART doses occur primarily on drinking days [17]. In keeping with these findings, the consumption of alcohol has been shown to be significantly associated with time to ART treatment failure as well as subsequent survival [18].

At a molecular level, in vitro experiments have demonstrated that exposure to even a moderate dose of alcohol can increase HIV replication in peripheral blood mononuclear cells [19]. Evidence suggests that alcohol use may lead to an increase in HIV viral replication in those infected with HIV [19–23]. Alcohol consumption may also impact CD4 cells, with research demonstrating lower CD4 counts among heavy alcohol users [24–26].

Despite the demonstrated links between alcohol consumption and HIV, few randomised controlled trials (RCTs) have evaluated the efficacy of alcohol-focused interventions in the context of HIV treatment. Furthermore, when such interventions have been implemented, the results have been equivocal [27–30]. There is considerable evidence from systematic reviews of RCTs that brief interventions (BI) are effective for reducing the volume of alcohol consumed and hazardous/harmful alcohol use among a range of patient populations, including those receiving primary health care [31–33]. The brief interventions (BI) we have chosen are based on motivational interviewing (MI) techniques and problem-solving therapy (PST), a variant of cognitive-behavioural therapy (CBT). Both of these modalities have proven efficacy for reducing alcohol consumption and problem drinking among a range of patient populations [34–38]. In the proposed study, we will bolster the effect of MI on hazardous and harmful drinking and improve the durability of MI’s effects by blending MI techniques with core elements of PST. Similar to MI, there is a large body of evidence in support of the efficacy of CBT for reducing problem drinking [38–41]. While the elements of this blended intervention are based on well-established treatment modalities of proven efficacy, they have not been implemented among PLWHA in South Africa. However, findings from our earlier work suggest that this blended intervention is likely to be acceptable to this population [42].

The overall goal of this project is to improve knowledge on the efficacy of an alcohol reduction intervention on alcohol consumption among PLWHA on ART, ART adherence and HIV treatment outcomes. The study has the following specific aims:

(i) *To adapt a blended MI and PST intervention to address harmful/hazardous alcohol use in HIV positive populations in Tshwane, South Africa*.
(ii) *To assess the efficacy of the blended MI-PST alcohol-focused intervention for reducing the average volume of alcohol consumed, improving ART adherence, maintaining ART adherence, improving HIV treatment continuation, and reducing disease progression relative to an equal-attention wellness intervention and treatment as usual (TAU) control group*. We hypothesise that those patients who receive the MI-PST alcohol-focused intervention will be more likely than those in the wellness intervention or TAU control groups to reduce their volume of alcohol consumption (our primary outcome) and that the participants in the alcohol-reduction intervention who reduce their alcohol consumption will have significantly greater improvements in their degree of ART adherence and in their HIV disease-related outcomes (including their treatment continuation and the progression of their disease) relative to participants in the wellness intervention or control group.
(iii) *To qualitatively assess counsellors’ and participants’ subjective responses to the blended MI-PST intervention*.

## Methods/Design

The proposed project will comprise three phases corresponding to the three specific aims. In Phase 1 (Aim 1), we will adapt an existing blended MI-PST intervention for use among patients receiving treatment for HIV. Phase 2 (Aim 2) will entail an RCT to assess the efficacy of this blended intervention in reducing alcohol consumption and in turn, improving levels of adherence to ART and HIV disease outcomes among ART patients in clinical care. Phase 3 (Aim 3) will involve a process evaluation to assess the counsellors’ and participants’ perceptions of the acceptability and impact of the alcohol intervention.

### Phase one: adapting MI-PST to reduce alcohol use among PLWHA initiating ART

We will adapt and refine our MI-PST intervention materials for use among patients receiving HIV disease care through conducting four focus group discussions (FGDs) with up to eight patients per group. They will discuss their perceptions about (1) how alcohol use relates to ART adherence; (2) potential barriers and facilitators to patient uptake of the proposed interventions; and (3) the acceptability, appropriateness and relevance of the proposed interventions. Each group discussion will be audio-taped and transcribed. Participants will receive refreshments during the FGDs, an incentive (a supermarket voucher worth ZAR50, approximately US$4.76), and ZAR50 for public transport to the clinics.

Once the intervention has been initially adapted, we will train health counsellors to pilot the blended MI-PST intervention for alcohol use and the MI-PST intervention for the wellness intervention. This pilot will assess the feasibility and acceptability of conducting these interventions among PLWHA in clinical care settings and test the intervention materials and procedures prior to scaling up for the RCT. Up to 20 patients who meet the eligibility criteria will be requested to provide informed consent prior to assessment and participation in the intervention. During the assessment, we will pre-test the study’s battery of questions to get a sense of how long it would take to complete the assessment as well as whether there is a need to revise any parts of the questionnaire.

In addition, we will pre-test the procedures for collection of biological specimens from the participants in order to assess their HIV viral load and CD4 T-cell counts, and Phosphatidylethanol (PEth), a biomarker indicative of recent alcohol use. Participants will then receive either the 4 session blended MI-PST alcohol reduction or the equal attention MI-PST wellness intervention over two days (Sessions 1 and 2 in Day 1, and Sessions 3 and 4 in Day 2).

Following the final intervention session, they will be re-interviewed to assess their perceptions of the factors that helped and hindered behaviour change. These interviews will be audio-taped and transcribed and used to further refine the intervention materials prior to their use in the RCT. Participants will receive an incentive after completing each intervention session and for completing the follow-up interview. Equal effort will be put into developing and adapting the alcohol and wellness interventions.

### Phase two: randomised controlled trial

#### Design

**Figure 1:**
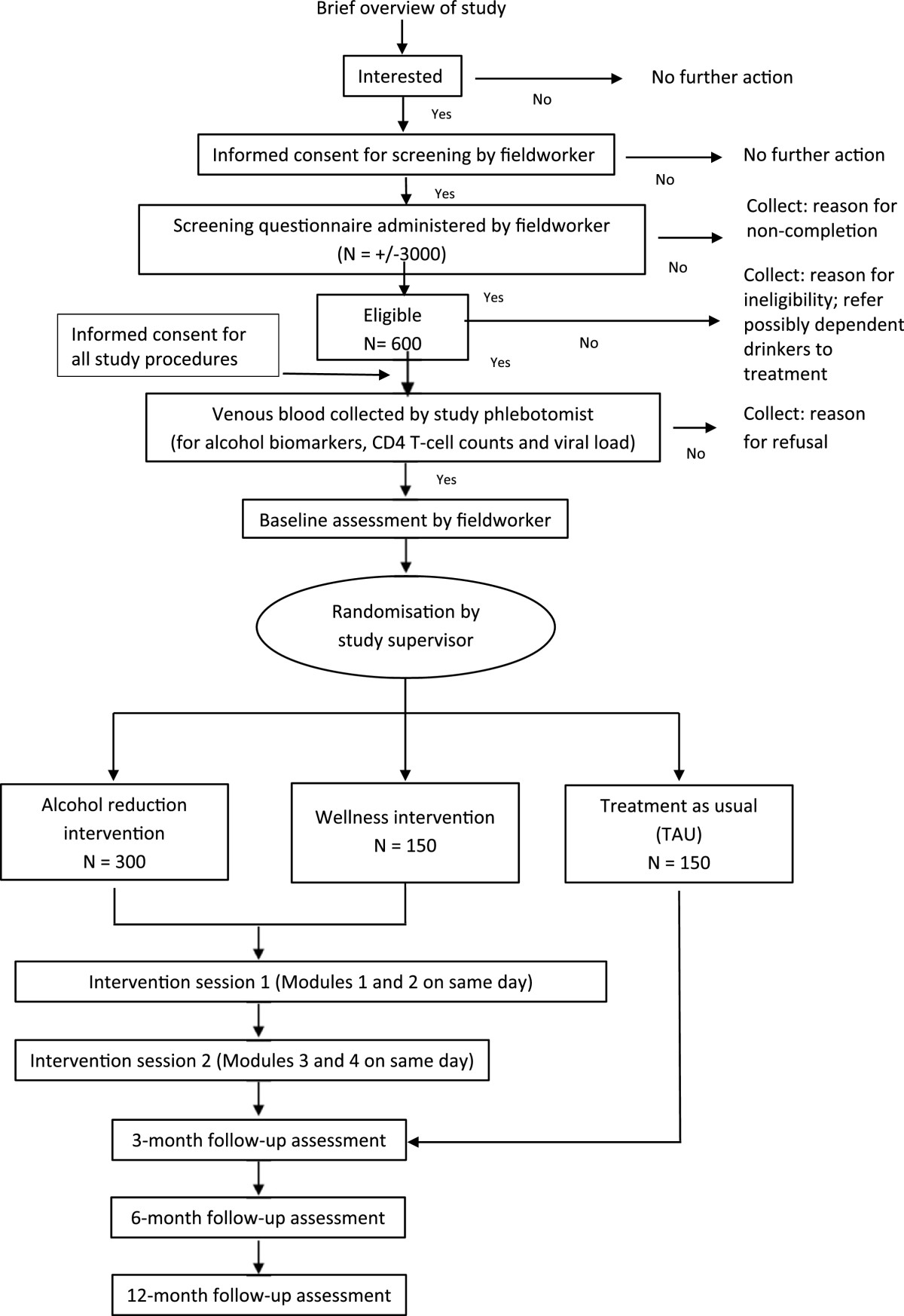
**Consort diagram of the study design.**

The proposed parallel, individual RCT will use a repeated measures design (see Figure 1, Consort diagram). The study will be a three-arm comparison, with one pre-intervention observation and three repeated measures post-intervention observations per participant. Randomisation to interventions will be done within sites.

#### Study population and sample size considerations

All participants will be recruited from ART clinics in hospitals in the Tshwane Health District. The inclusion criteria for the clinics include having funds for the duration of the project so that ART supplies do not run out, being located within the Tshwane Health District, and having a large active caseload of PLWHA receiving ART (e.g. at least 100 per week) to facilitate patient recruitment. Individuals will be eligible to participate in this research if they (1) are HIV-positive; (2) have taken ART for at least three months; (3) are not currently receiving treatment for TB (or latent TB infection); (4) are 18 years or older; (5) are classified as “harmful/hazardous drinkers” (have six (or four, if female) or more drinks on one occasion at least weekly, and score no more than 22 on the AUDIT); (6) are resident in the Tshwane Metropolitan Municipality or the general area served by the clinic/site; (7) have no substantial cognitive impairment; (8) are not currently enrolled in another trial; and (9) do not have extremely poor general health/functional status (Karnofsky clinical score > 50).

Sample size estimates are based on the numbers of patients needed to detect differences between intervention and control groups in the mean number of drinks per day (past 30/60 days) at 3, 6, and 12 months post randomisation, our main outcome measure. A study among HIV patients found that the mean number of drinks per drinking days was 3.58 (Standard Deviation (SD): 1.81) under an enhanced MI vs. 4.75 (SD: 3.22) under the control arm [43]. For two-sided testing at a significance level of 5% and power of 90%, with one pre- and three post- intervention measures with a within subject correlation of 0.7 between measurements, the required sample size using analysis of covariance methods is 260. Of the 260 participants, 130 will be allocated to the alcohol intervention and 65 to each of the wellness and TAU arms. This imbalance of sample sizes for the treatment combinations can cause loss of power of statistical tests of differences between the group outcomes (e.g. means). It may also produce sensitive results in small samples when the assumptions of equal variance between groups are violated. Nonetheless, our choice of the imbalanced design was based on valid scientific and practical reasons, as the intervention combination we plan to assess is of great interest. Thus, the higher sample size in the main intervention arm (alcohol intervention) was chosen as it is statistically more efficient to have more subjects in this group as we will be comparing its effect to each of the other two interventions. This sample size also allows adequate power (>90%) to detect a modest effect size (0.25) for the within-subjects component, and for a potential interaction between- and within-subjects factors. However, the actual number of participants needs to be inflated to take into account attrition over time. We estimate an overall attrition rate of 20% and therefore need to recruit 325 participants to take into account this attrition. Assuming four sites, this would create a site-specific sample size of 82 (66 after attrition).

We have no site-specific hypotheses, but if we want to include a safety margin for testing site specific effects of 0.30, we should allow for a site specific sample of 150 which would already include the attrition (120 per site before attrition). Of these, 75 would receive the alcohol intervention, and 38 each the wellness intervention and TAU. We will recruit participants from each site without an attempt to have equal numbers of men and women; we anticipate a ratio of about 2:1 females to males. Based on our prior data [44], we would estimate that about 40% of ART recipients will be alcohol drinkers. Of the alcohol drinkers, we estimate that about 15% would meet the criterion related to alcohol (have six (or four, if female) or more drinks on one occasion at least weekly, and score no more than 22 on the AUDIT). That research also suggests a participant eligibility rate of no more than 20%, so we would need to screen at least 750 patients at each site to reach our target sample of 150. Therefore we need to recruit participants from large facilities, suggesting that only hospital-based clinics would qualify as possible study sites. Thus, we plan to recruit participants from all four of the district hospitals in the Tshwane Health District, which cover a wide catchment area. The hospitals are: (1) Tshwane District Hospital, (2) Pretoria West Hospital, (3) Odi District Hospital, and (4) Jubilee Hospital. With four sites, our final sample size of 600 will include 300, 150 and 150 participants in the alcohol, wellness and TAU arms respectively. In the proposed district hospitals, there are HIV clinics in the form of separate outpatient departments (OPD). These HIV clinics carry very high volumes of patients, spanning the whole continuum of care.

#### Recruitment procedures

Within each study clinic, the research staff will identify HIV-positive participants who have been on ART and provide them with a brief overview of the study. Interested individuals will then be referred to a fieldworker, who will request informed consent for the completion of a brief screener for assessing whether or not they meet the eligibility criteria listed above. With one of the proposed inclusion criteria being classified as a “harmful/hazardous drinker” (have six (or four, if female) or more drinks on one occasion at least weekly, and score no more than 22 on the AUDIT), those with AUDIT scores in the range of possibly dependent (>22) will not be eligible for the study, hence they will not get to the randomisation stage. Once the participant has consented to take part in the study he/she will be assigned a random ID number which will determine the condition into which he/she will be placed: the alcohol-reduction intervention condition, the wellness intervention condition, or the TAU condition. Consenting individuals will then be asked to provide a sample of just under 25 ml of venous blood, to assess biomarkers that are indicative of recent levels of alcohol consumption and assess their HIV viral load and CD4 T-cell counts. The blood samples will be drawn by a registered nurse or phlebotomist in a private space and transported to a local laboratory for testing. The participants will then be interviewed by a fieldworker on their demographic characteristics, alcohol-related issues, ART adherence, and health-related issues. The interviews will be conducted by a trained interviewer who will be independent of the interventionists and clinic staff. Participants assigned to the alcohol reduction and wellness conditions will then be asked to return within one week (or two weeks, if not available during the following week) to receive their first and second intervention sessions, and return after another week, to receive their third and fourth intervention sessions. All participants, irrespective of condition assignment will be asked to return at three, six and twelve months’ post-randomisation to complete follow-up assessments (interviews and further biological testing). Participants’ transport costs will be covered for each contact with project staff.

#### Randomisation and blinding

Randomisation to one of the three conditions will occur at each of the four sites, i.e. the randomisation will be stratified by site. The randomisation code will be generated by computer generated tables. Treatment allocation will be done by site supervisors using pre-prepared consecutively numbered sealed, opaque envelopes, which will contain the group assignment. Fieldworkers will conduct the post-intervention outcome assessments masked to treatment allocation to ensure that the assessments remain unbiased and independent from the intervention sessions. Each counsellor will take part in only one intervention arm, and the wellness and alcohol reduction interventions will be run on different days of the week (based on alternating biweekly schedule) to minimise contact between participants who are randomly allocated to different arms, and to minimise contamination across the study arms. Given the study’s behavioural nature, the interventionists will not be blinded to which intervention the participants receive.

#### Intervention conditions

##### Condition 1: Blended MI and PST for alcohol

The overall goals of this blended intervention are to motivate participants to reduce their hazardous or harmful drinking and equip them with problem-solving skills that will improve their ability to cope with alcohol use triggers. This experimental condition will comprise four modules conducted over two sessions, with these sessions spread one week apart (Table 1). These four sessions allow us to cover all of the core principles of PST and allow time for practicing of skills. In formative research to determine the appropriateness and acceptability of our MI-PST intervention for patients attending primary health care services, one hour was the most preferred session length, and the vast majority of patients were willing to return to the clinic for two to three extra visits. Hence we have tailored this intervention to meet patients’ preferences [45]. Each session iteratively builds upon participants’ readiness to change and the problem-solving and coping skills developed in the previous session, and has a motivational, educational and practical component. The sessions’ structure conforms to our previous research observations that ART recipients most preferred one-hour long sessions, and two-session interventions.

**Table 1:** **Blended motivational interviewing (MI) and problem solving therapy (PST) intervention**

	Summary of blended MI and PST sessions and objectives
**Session 1, Week 1** 45 minutes	• Conduct screening/assessment of alcohol use
	• Provide feedback on results of screening/assessment
	• Increase knowledge of how alcohol use impacts on course of HIV
	• Use MI to build rapport and develop readiness to change
	○ Assess readiness to change (using readiness ruler)
	○ Assess pros and cons of change (Decision-balance exercise)
	○ Use MI to try and shift participant and elicit a commitment to change
**Session 2, Week 1**	Patient check-in (using MI)
50 minutes	• Build the Rationale for PST
	• Explain the structure of PST
	• Explain the link between problems and alcohol use, and the rationale for PST
	• Establish positive problem orientation
	• Describe the steps of PST
	• Build the rationale for activity scheduling
	• Describe the steps of Problem Solving
	• First Problem Solving Session with counsellor (using the steps) and describe homework
**Session 3, Week 2** 50 minutes	• Patient check-in (using MI)
	• Review homework from previous week and challenges/difficulties
	○ Elicit positive change talk and affirm commitment to change using MI techniques
	○ Review PST steps and affirm attempts to change
	• Explain what can be done about problems that are not important (coping with negative thoughts)
	• Second Problem Solving Session with counsellor and an exercise
**Session 4 Week 2** 45 minutes	• Review practice exercises from session 3
	• Explain what can be done about problems that are important but cannot be solved 5 min (advancing the process of acceptance)
	• Third Problem Solving Session with counsellor and recap
	• Elicit positive change talk and affirm commitment to change using MI techniques

##### Condition 2: Blended MI-PST for wellness

The wellness intervention will be an equal attention-control arm. Like the alcohol reduction intervention, this intervention will incorporate MI-PST techniques to promote reduction of health risk behaviours, healthy nutrition and a healthy lifestyle. It will be delivered across four sessions, with Sessions 1 and 2 occurring in Week 1 (separated by a break) and Sessions 3 and 4 occurring in Week 2. Participants will be taught about healthy eating, to maintain a healthy weight, and the value of exercise for optimum health.

##### Condition 3: Treatment as usual (TAU)

Participants in this condition will receive the standard package of care for PLWHA and on ART who also drink at hazardous/harmful levels. Typically, if a health care provider suspects adherence problems (by self-report or clinical indicators), the patient is referred to adherence counsellors for additional counselling. There are no firm guidelines on the duration or content of this counselling. Counsellors are left to determine how best to address adherence problems with the patient. If adherence counsellors or primary health care providers suspect alcohol problems, they usually refer patients to on-site clinical psychologists/clinical social workers, if available, or off-site community mental health (CMH) or alcohol services. We will monitor the number of visits the TAU patients make for adherence and alcohol support, and type(s) of support services received (e.g. advice, referral) by means of self-report at the 3, 6, and 12 month follow-ups.

#### Follow-Up

Participants will be followed-up on three occasions: at three, six and 12 months (Table 2). At each follow-up visit, they will be interviewed using a structured questionnaire. They will have blood drawn for the following tests: viral load (at baseline and 12-month follow-up); CD4-T cell count (at each follow-up point); and alcohol biomarker PEth (at each follow-up point). Compensation will be provided to participants in all arms in the form of shopping vouchers for completion of the follow-up assessments.

**Table 2:** **List of study instruments, data elements and source of information**

Data collection instruments	Time period	Data collected	Source
Screening questionnaire	Day of enrolment	Eligibility assessment	Self-report
Participant assessment	Baseline 3, 6, and 12 month follow up	Demographic information, alcohol consumption, social problem solving styles, motivation/readiness to change, antiretroviral therapy (ART) history, adherence to ART, psychosocial factors associated with ART adherence (disclosure, HIV/AIDS stigma, social support, adherence self-efficacy), alcohol and ART beliefs, HIV-related factors (e.g. degree of ill-health between HIV diagnosis and ART initiation), clinical/structural factors (e.g. length of time to doctor), physical functioning (e.g. limitations due to physical health, bodily pain, vitality), general physical well-being, mental well-being, drug and tobacco use	Self-report
Laboratory report form	Baseline, 3, 6, 12 month follow up	HIV-related outcomes: viral load (baseline and 12-month follow up assessments only) and CD4 counts	Laboratory report
		Alcohol biomarkers: Posphatidylethanol (PEth)	
Process evaluation interview schedule	12-months follow-up	A randomly selected 10% subsample will respond to a qualitative interview regarding impressions of the degree to which the intervention helped or hindered behaviour change	Self-report

#### Participant retention

In order to enhance participant retention we will: (1) inform interested individuals at recruitment that participation in the study will last for 12 months, and that if they anticipate relocating to another area or clinic they will not be eligible to participate. (2) Ask participants to provide their own and two close relatives’ or friends’ contact details, including telephone numbers and physical addresses to facilitate regular contact with them, reminder calls, and other participant tracking efforts.

#### Measures

We will use a structured questionnaire at baseline and each follow-up period. It will be available in English, Afrikaans and seTswana. We will assess the following:

- *Demographic Characteristics.* We will assess participants’ age, gender, race/ethnicity, income, education, employment status, housing status, relationship status, sources of income and food insecurity.
- *Alcohol Consumption.* Multiple self-report measures will be used to assess various dimensions of alcohol consumption. The Alcohol Use Disorders Identification Test (AUDIT) [46] will be employed as part of the initial screening process, as well as all further follow-up assessments to identify potential harmful and hazardous drinking. We will change the reporting period of the AUDIT from 12 months to 3 months at baseline and at each of the follow-up periods. We will only ask participants to respond to the AUDIT questions for the past 12 months during the screening. Additional items will assess current drinking/abstention (past 90 days, past 12 months and lifetime). The 3-month quantity item will serve as the primary behavioural outcome measures for alcohol consumption, together with a combined quantity-frequency measure.
- *Social Problem Solving Styles.* We will employ the 25-item Social Problem-Solving Inventory Revised-Short Form (SPSI-R: SF) [47], which measures problem solving styles. Previous South African studies have obtained Cronbach’s alphas of 0.72-0.83 for this scale [48].
- *Motivation/Readiness to Change.* The participants will be asked to respond to the 12-item Readiness to Change Questionnaire to determine their readiness to change their drinking behaviour [49].
- *Adherence to ART.* Four adherence measures will form the basis of the self-reported ART adherence outcomes: (1) The AIDS Clinical Trials Group (ACTG) adherence questionnaire; which assesses patients’ current ART medications, dosing schedule, and medication doses missed over the past four days [50]; (2) The Visual Analog Scale (VAS), which assesses general levels of adherence over a 30-day timeframe [51]. For the ACTG and VAS, adherence levels will be examined in terms of both continuous and dichotomous measures (e.g. cut-offs of 90%, 95%, and 100% adherence rates); (3) The CASE Adherence Index [52]; and (4) The Self-Rating Scale Item (SRSI) [53, 54].
- *Health Functioning.* The SF-8 is an eight item measure of health and well-being, assessing physical functioning, limitations due to physical health, bodily pain, general health, vitality, social functioning, limitations due to emotional health, and mental health issues [55].
- *Mental Well-Being.* The Kessler Psychological Distress Scale-10 [56] will be used to assess psychological distress.
- *Drug and Tobacco use.* The Drug Use Disorders Identification Test (DUDIT) [57] is an 11-item self-report measure that will be used to assess concurrent substance use. Participants will also be assessed for tobacco use (including lifetime, current and regular cigarette smoking) and tobacco dependence using the Fagerstrom Test for Nicotine Dependence (FTND) [58].
- *Alcohol Biomarkers.* In addition to the use of self-report measures of alcohol use, tests of the alcohol biomarker, Phosphatidylethanol (PEth) [59, 60] will be conducted among all participants at each time point.
- *Clinical/Structural Factors*. The following structural factors typically associated with ART adherence will be assessed: Time to doctor; difficulty getting to the clinic. These are reasons for difficulty getting to the clinic. Based on previous research with HIV clinic patients in South Africa [61].
- *HIV-Related Factors*. The following information will be obtained: perceived year and month of diagnosis; perceived current HIV viral load and CD4 cell count, advent of opportunistic infections, the degree of ill health between diagnosis and starting on ART, and the duration from diagnosis to ART enrolment.
- *ART History*. We will assess the duration of being on ART and duration on the current regimen.
- *Psychosocial Factors*. Psychosocial factors typically associated with ART adherence will be assessed. For example, HIV stigma will be measured with the Internalised AIDS-Related Stigma Scale [12], HIV disclosure will be assessed via a measure used in our prior studies [42, 44], social support will be assessed by the MOS Social Support Survey [62], and adherence self-efficacy will be assessed by the HIV Treatment Adherence Self-Efficacy Scale (HIV-ASES) [63].
- *General Physical Well-being*. The Karnofsky Performance Scale will be used to evaluate the general health/functional status of prospective participants. Scores range from 0 to 100; the lower the score, the more compromised general well-being [64, 65]. Individuals with a Karnofsky clinical score below 50 will not be eligible for participation.
- *Alcohol and ART*. We will ask about (1) the source(s) of participants’ knowledge about alcohol use and ART; (2) beliefs about alcohol and ART; (3) beliefs about the acceptability of drinking while on ART; and (4) patterns of drinking while on ART.
- *Screening for Cognitive Impairment*. We will use the International HIV Dementia Scale (IHDS) [66] to screen for substantial cognitive impairment. The cut-off score on this instrument is 10.

### Phase 3: process evaluation

Following the final follow-up assessment, we will randomly select 10% of participants (from the wellness and MI-PST conditions) for qualitative in-depth interviews. These interviews will assess participants’ impressions of the degree to which they felt the intervention helped or hindered behaviour change. They will be conducted by project staff not involved in the assessments or interventions in order to minimise response bias. The qualitative interviews will be conducted in the participants’ language of choice. All interviews will be audio-taped and transcribed verbatim. Findings from these qualitative interviews will be used to interpret and better understand the quantitative findings from the RCT. Participants will receive an incentive for their time and participation in these interviews and a voucher to cover transport costs.

### Study timeline

The first nine months of the study will involve intervention and measurement development, piloting of intervention components and training field staff and counsellors. Study recruitment will be conducted at the end of Year 1 and during the first 3 months of Year 2. If necessary, timelines may be adjusted based on information gained during the pilot phase. Intervention sessions will commence in the last quarter of Year 1 and continue during the first half of Year 2. Three and six month follow-up assessments will be conducted in Year 2. Twelve-month follow-up assessments will be conducted in Year 3. Quarters 3–4 of Year 3 will involve data synthesis, analysis, write-up and dissemination.

### Statistical treatment and analysis of data

#### Analyses for phase 1

All audiotapes will be transcribed into MS Word. MS Word documents will be imported into Nvivo 10 [67]. Data analysis will be conducted using the framework approach (familiarization, identifying a thematic framework, indexing, charting, mapping and interpretation). Initially, transcripts will be read for emergent themes, which will then be coded. Coding and analysis will continue iteratively as new themes and issues emerge; all relevant information will be retrieved and examined for further coding designations. To establish inter-coder reliability, each transcript will be coded by at least two individuals. Coding discrepancies will be discussed and resolved and transcripts will be subjected to checks until the Cohen’s Kappa score reaches 70% agreement. We will use these findings to assist with revisions to our proposed intervention materials and implementation protocols.

#### Analyses for phase 2

Given the repeated measures design, the main statistical analysis will involve repeated analysis of variance and analysis of covariance, using pre-treatment means and post-treatment means; or two-sample t-tests or chi-squared tests at the patient level data. The hypothesis that alcohol reduction will increase adherence will be tested via a repeated-measures Analysis of Variance (ANOVA). This will be an intent-to-treat (ITT) analysis. For our ITT analysis we will analyse our data based on the participants’ original allocation to the groups regardless of any subsequent group changes or participants’ degree of adherence to study protocols or their withdrawal from the study. We will assume data will be missing completely at random (MCAR) in order to enable imputation of missing data using standard techniques. We have postulated a main effect for the experimental condition without an interaction effect. In addition, we intend to use the CD4 T-cell counts and viral loads as objective biological endpoints, with the same statistical procedure being employed. Additional analyses will determine whether the pathway via reduction of alcohol consumption was indeed crucial to the differences in adherence. This can be measured by a cross-lagged panel design, where the alcohol in the time interval before the respective measurement of adherence will be assumed to have a significant effect. Structural equation modelling and path analysis will be used to test this assumption. Models will also be run with ART continuation as the endpoint. More advanced techniques, including generalised estimating equation and multilevel modelling, will also be employed. The association between the biomarker of alcohol consumption and self-reports of alcohol will be analysed using correlation and regression techniques with adjustments for possible confounders. The predictive ability of the biomarker will be assessed using sensitivity and specificity techniques to determine its ability to detect excessive self-reported consumption of alcohol.

#### Analyses for phase 3

Data analysis will be conducted using the same framework approach as described above for Phase 1. We will use the Phase 3 findings to assist with interpreting the findings of the RCT phase.

### Ethics

Paper-based data will be stored in locked filing cabinets at the MRC offices in Pretoria for five years. Study data and consent forms will always be stored apart from each other in separate locked filing cabinets. Electronic data files will be encrypted, password protected and saved onto secure computers. Samples will be identified by their study identification number and will not include participants’ names.

In order to secure quality control and quality assurance of the study, research staff and counsellors will receive training in research ethics for human subjects. Second, a data manager will be employed to ensure accurate data collection; the maintenance of participant confidentiality; and the secure transfer and storage of electronic data. Third, weekly feedback meetings will be held among study personnel, the data manager, and the project manager and will address possible problems. Separate weekly meetings will be held with the project manager and PIs. Fourth, a Data and Safety Monitoring Board (DSMB) will review study-related concerns (e.g. adverse events) that may arise and will meet six-monthly. Finally, independent study monitors will review study protocols and procedures (e.g. data collection and storage), at two points during the data collection phase.

Safeguards will be implemented throughout the project to protect participant confidentiality, as described in the Human Subjects Protection Plan section, and to minimize the risk of potential physical and psychological harms. All procedures will first be pilot tested for acceptability with a small sample of individuals from the target population, and any study-related activities identified as harmful or as a cause of discomfort will be amended.

Ethical approval for this study has been obtained from the Medical Research Council’s Ethics Committee. Approval to conduct the study has also been obtained from the Regional and Provincial Departments of Health, and the site managers of each clinic. Participants will be informed that their decision to participate in the study is voluntary and will not affect their current or future care at the clinic. They will also be informed that even after deciding to participate in the study, they can still decline to answer questions or to take part in specific activities, and they may terminate their participation at any time without any penalty. Informed consent will be obtained by research staff who are not clinic employees or connected to participants’ medical care. Through the informed consent process, prospective participants will be informed of all foreseeable risks involved in the study. Individuals with low or no literacy will have the consent form read to them by a person of their choosing, and verbal consent will be obtained, witnessed by an independent person. The consent forms will be translated and back-translated and adapted for cultural appropriateness and readability into SeTswana and seSotho, the main local languages of the region. To ensure confidentiality, study data and biological samples will be identifiable only by randomly generated participant identification numbers and will not contain participants’ names or any other personal identifying information or protected health information. All electronic data sources will be encrypted and password protected. Research personnel who have contact with participants will be asked to sign a confidentiality agreement, and receive on-going training and supervision on ethical conduct and confidentiality protection. To minimise the possibility of a breach of confidentiality by participants during the focus group discussions, the participants will be asked to not disclose the identity or the content of the commentary of any of their fellow focus group discussion members to anyone outside of the focus group.

Overall, only minimal risks are anticipated. Some participants may feel uncomfortable when answering the assessment questions which address sensitive topics (e.g. alcohol use; non-adherence); and/or when discussing their alcohol consumption with the intervention counsellors; and/or when providing blood samples. There are potential benefits to study participants. Any individual identified as alcohol dependent will be referred for alcohol treatment services. Individuals assigned to the alcohol reduction condition are expected to reduce their alcohol consumption, and in turn improvements in their level of ART adherence, and their HIV disease course. Moreover, at the end of the intervention period clinic staff (and/or other suitable health workers) will benefit as they will be trained on delivering the intervention aimed at reducing harmful/hazardous alcohol consumption. Finally, there is substantial public health knowledge to be gained from this research.

## Discussion

We hypothesize that compared to individuals who receive the wellness-focused BI, those who receive the alcohol-focused BI will demonstrate greater reductions in alcohol consumption, and as a result, they will demonstrate higher levels of ART adherence and higher CD4 counts, and will be less likely to have detectable HIV viral loads. This study will indicate whether alcohol-focused interventions are feasible to implement with clinical HIV populations, and also whether they can be used to successfully improve adherence to ART treatment and health-related outcomes among people being treated for their HIV infection.

The proposed study has the potential to advance scientific knowledge in several ways. First, it will be among the first to use a blended MI and PST intervention to target reductions in alcohol use among PLWHA in clinical settings in South Africa. While evidence of the link between alcohol use and poor adherence to ART has led for calls for behavioural interventions to reduce risky drinking among PLWHA, there have been few interventions implemented in clinical settings to address this risk for poor HIV disease outcomes. This study will not only help us answer questions about whether reducing hazardous and harmful alcohol use will lead to improved adherence and HIV disease outcomes but will also provide knowledge about what kinds of behavioural interventions are effective for reducing hazardous/harmful alcohol use among PLWHA in clinical care.

Second, this study hopes to further explain the relationship between alcohol use, ART adherence and HIV disease outcomes. If the proposed intervention is effective, we hope to find that reduced alcohol use facilitates better adherence to ART and consequently improved disease outcomes. This would be a significant contribution to health knowledge and could lead to the development of clinical guidelines for the management of alcohol use among people living with HIV/AIDS. Third, through the employment of alcohol and HIV disease-related biomarkers to assess intervention efficacy, this study will be at the forefront of research conducted in the area of alcohol reduction and ART adherence promotion, and will provide considerable objective data regarding the links between alcohol consumption, ART adherence, and health outcomes.

Finally, testing the proposed intervention in clinical settings is expected to yield valuable information about how best to introduce and implement the proposed intervention into clinical services. This information will help lay a solid foundation for future studies that examine how best to integrate behavioural interventions for alcohol use and other psychosocial risks for poor ART adherence and HIV disease outcomes into HIV treatment services. Taken together, results from this work could have far-reaching implications for the development and implementation of clinic-based programmes as well as broader policies designed to reduce alcohol consumption, which in turn, can help improve the health and well-being of those living with HIV/AIDS.

Any individual identified as alcohol dependent during the screening process or during the course of the study will be provided with a referral for alcohol treatment. For individuals taking part in the study who are assigned to the active treatment condition (MI/PST), it is anticipated that the alcohol counselling that is provided will not only help to reduce their alcohol consumption, but it may also help them improve their level of adherence, which in turn could help decrease the likelihood of worsening the course of their HIV disease and the acquisition of opportunistic infections. With improvements in ART adherence stemming from the intervention, participants should also experience reductions in their HIV viral load. This, in turn, can have a significant public health benefit, whereby this decreased infectivity can result in a reduced likelihood of participants passing on HIV to their partners through unprotected sexual contact [68, 69]. Finally, should the intervention be found to be effective, we will offer training to existing staff at the four clinics (and/or other suitable health workers) in how to screen and identify individuals whose drinking may be detrimental to their health and also in how to deliver psychosocial interventions aimed at reducing harmful and hazardous alcohol consumption.

## Authors’ original submitted files for images

Below are the links to the authors’ original submitted files for images. Authors’ original file for figure 1

## Abbreviations

ACTG: AIDS Clinical Trials Group
AOD: Alcohol and other drug
ANOVA: Analysis of Variance
ART: Antiretroviral therapy
AUDIT: Alcohol use disorders identification test
BI: Brief intervention
CBT: Cognitive-behavioural therapy
CMH: Community mental health
DUDIT: Drug use disorders identification test
FTND: Fagerstrom test for nicotine dependence
HIV: Human immunodeficiency virus
HIV-ASES: HIV treatment adherence self-efficacy scale
IHDS: International HIV dementia scale
ITT: Intent to treat
MI: Motivational interviewing
OPD: Outpatient department
PEth: Phosphatidylethanol
PLWHA: People living with HIV and AIDS
PST: Problem solving therapy
RCT: Randomized controlled trial
RNA: Ribonucleic acid
SRSI: Self-rating scale item
SPSI-R: SF: Social problem-solving inventory revised-short form
TB: Tuberculosis
TAU: Treatment as usual
VAS: Visual analog scale.
