# Supplementary figures and images for: Efficacy of an alcohol-focused intervention for improving adherence to antiretroviral therapy (ART) and HIV treatment outcomes – a randomised controlled trial protocol
Source: BMC Infect Dis. 2014 Sep 12;14:500. doi: 10.1186/1471-2334-14-500 (PMC4174635; doi:10.1186/1471-2334-14-500)

Brief overview of study

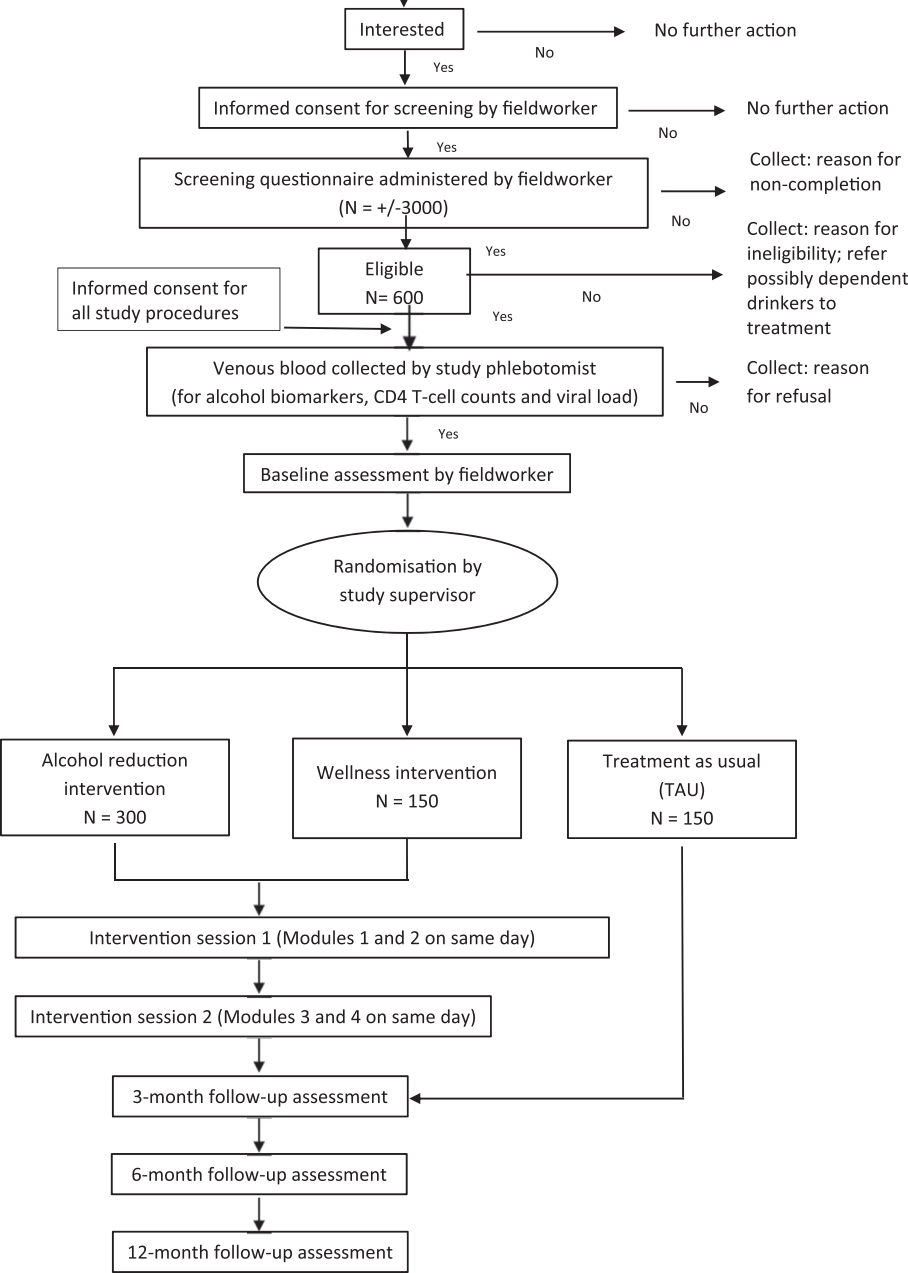

Supplement: Supplementary file 1 — Authors’ original file for figure 1 [file 12879_2014_3804_MOESM1_ESM.pdf]
